# Biomarkers of severe dengue disease – a review

**DOI:** 10.1186/s12929-015-0191-6

**Published:** 2015-10-14

**Authors:** Daisy Vanitha John, Yee-Shin Lin, Guey Chuen Perng

**Affiliations:** Biotechnology Research Institute, University Malaysia Sabah, Kota Kinabalu, Sabah Malaysia; Center of Infectious Disease and Signaling Research, Department of Microbiology and Immunology, College of Medicine, National Cheng Kung University, Tainan, Taiwan

**Keywords:** Severe dengue, Biomarkers, Immune activation, Endothelial activation

## Abstract

Dengue virus infection presents a wide spectrum of manifestations including asymptomatic condition, dengue fever (DF), or severe forms, such as dengue hemorrhagic fever (DHF) and dengue shock syndrome (DSS) in affected individuals. The early prediction of severe dengue in patients without any warning signs who may later develop severe DHF is very important to choose appropriate intensive supportive therapy since available vaccines for immunization are yet to be approved. Severe dengue responses include T and B cell activation and apoptosis, cytokine storm, hematologic disorders and complement activation. Cytokines, complement and other unidentified factors may transiently act on the endothelium and alter normal fluid barrier function of the endothelial cells and cause plasma leakage. In this review, the host factors such as activated immune and endothelial cells and their products which can be utilized as biomarkers for severe dengue disease are discussed.

## Introduction

Dengue is a mosquito borne viral infection found in tropical and sub-tropical regions of the world and is caused by one of the four serotypes of dengue viruses (DENV1-DENV4). An increase in infection has been seen in recent years due to many factors including urbanization and air travel. Over 2.5 billion people of the world’s population are now at risk for dengue. The consequences of DENV infection range from asymptomatic condition, dengue fever (DF), or severe forms, such as dengue hemorrhagic fever (DHF) and dengue shock syndrome (DSS). Severe dengue is characterized either by plasma leakage, fluid accumulation, respiratory distress, severe bleeding, or organ impairment [1]. Clinical manifestations offer the earliest markers in predicting severe dengue disease. A recent meta-analysis of signs and symptoms of severe dengue shows that bleeding, nausea and vomiting, abdominal pain, skin rashes, and hepatosplenomegaly are associated with severe dengue disease [2]. Patients with dengue fever are clustered into two groups: one with warning signs including abdominal pain, mucosal bleeding and liver enlargement that warrant ICU admission and the other without those signs [1, 2]. Early prediction of severe dengue in patients without any warning signs who may later develop severe DHF is very important to give the best supportive care since approved vaccines for immunization are yet to be commercialized. An ideal biomarker should be able to identify individuals who are at risk of developing severe dengue.

The mechanism by which only a few DENV infected individuals progress to severe dengue disease is poorly understood. The host immune responses have been considered as the major factor responsible for dengue pathogenesis. The process of plasma leakage, shock and hemorrhagic manifestations initiated by enhancing infection with DENV virus with the help of opsonizing antibodies, resulting in an altered immune response which trigger T cell activation and release of cytokines and chemical mediators has been a risk factor in secondary infection [3, 4]. However, undefined factors could play a role in the development of severe dengue in individuals with naïve primary infection and immune non-responders [5]. Dengue patients show fever symptoms during peak of viremia while DHF/DSS appears during the time when the virus has been cleared from the circulation suggesting severe dengue disease is most likely associated with immunopathology. Thus, the host immune response components including cells, cytokines, complements and other cellular mediators can serve as biomarkers of severe disease [6, 7]. It is reported that the macro-morphology of endothelial lining remains intact while the functionality of the endothelial cells is altered by activation which leads to vascular permeability resulting in plasma leakage [8]. Therefore, endothelial activation markers such as expression of adhesion molecules and receptors can also serve as biomarkers of severe dengue disease [9, 10]. In this review, the various host immune and endothelial activation markers and biochemical and genetic markers are reviewed for their utility as potential biomarker of severe dengue disease.

## Immune activation markers as predictors of severe dengue disease

### Number and activation status of immune cells

DENV has been shown to infect a wide range of cells including dendritic cells (DCs), monocytes, lymphocytes, hepatocytes, endothelial cells (ECs) and mast cells *in vitro *[6]. Although the role of these cells in DENV infection remains less clear *in vivo*, activation of memory T cells resulting in cascades of inflammatory cytokines and other chemical mediators that trigger death of target cells through apoptosis is a critical element contributing to severe dengue [11]. DCs and macrophages are the primary targets of DENV infection [12, 13]. Both the absolute number and frequency of circulating myeloid DCs (mDCs) and plasmacytoid DCs (pDCs) were decreased early in acute viral illness in children but not in adults who subsequently developed DHF and decreased level of pDCs was associated with higher viremia levels [14, 15]. Activated DCs may contribute to vascular leak through the production of TNF-α, IFN-γ and matrix metalloproteases-2, 3 and 9 [16, 17]. Studies show that CD4 T cell, CD8 T cell, NK cell and γδ T cell counts were significantly decreased in DHF compared to DF early in the course of illness [18]. The CD8 T cells and NK cells from dengue patients displayed activation markers such as CD69, HLA-DR, CD38 and cytotoxic granule TIA-1 and cell adhesion molecules CD44 and CD11a during the acute phase [19, 20]. The decreased numbers of lymphocytes could be due to increased apoptosis of peripheral blood mononuclear cells observed during DENV infection evidenced by the presence of increased plasma levels of soluble CD95, a mediator of apoptosis, and down-regulation of the antiapoptotic protein Bcl-2 in these cells [21, 22]. Cross-reactive memory T cells that are highly activated with increased levels of cytokine producing capacity was observed in patients with acute dengue disease. Activation of cross-reactive low affinity T-cells results in copious amounts of cytokine and chemokine production such as IFN-γ, TNF-α, IL-1, IL-6, IL-8, IL-10, CCL2 (MCP-1) and CCL5 (RANTES) [23, 24].

Activation of mast cells and increased levels of urinary histamine, which is a major product of mast cells were observed in dengue patients and the levels correlated with disease severity [25]. Several data indicated that virus stimulated mast cells selectively produced and secreted a variety of mediators including chemokines, cytokines, lipid mediators, and granule associated products. Elevated levels of secreted CCL3 (MIP-1α), CCL4 (MIP-1β) and CCL5 were observed following infection of human mast cell lines [26, 27]. Substantial levels of tryptase and chymase were found in mast cells and these proteases are considered to be selective markers of mast cell activation. Plasma levels of both tryptase and chymase were increased significantly in DHF/DSS compared with DF. [28] Thrombocytopenia is one of the clinical hallmarks for dengue patients. There are many mechanisms leading to the depletion of platelets in affected subjects, these include direct infection of megakaryocytes by DENV as well as platelet destruction due to nonstructural protein 1 (NS1) binding and platelet-associated antibodies [29–31]. Thrombocytopenia is best used as a marker of severe disease particularly when it is <100,000 cells/c.mm and serve as an indicator of prognosis during the course of the disease [18, 32, 33]. Since thrombocytopenia is seen in both DF and DHF patients, a platelet count of 60,000 cells/c.mm serves as a better cut-off in identifying more severe cases [7].

### Increased levels of cytokines and chemokines

Patients with DHF/DSS present a ‘cytokine storm’, with high levels of circulating cytokines and chemokines. Therefore, serum cytokine and chemokine levels can serve as a laboratory tool for predicting severe disease. T cells, NK cells, monocytes, macrophages, hepatocytes and ECs have been shown to contribute to the increased production of cytokines and chemokines. Increased levels of IFN-γ, TNF-α, IL-1β, IL-4, IL-6, IL-7, IL-8, IL-10, IL-13, IL-15, IL-17, IL-18, macrophage migration inhibitory factor (MIF) and chemokines CCL2, CCL4, CCL5, and CXCL10 (IP-10) have been reported in patients with DHF when compared to DF. [34–38] Studies show that elevated levels of IL-6, IL-10, IFN-γ, MIF, and CCL-4 could be used as potential predictors of severe dengue [39–46].

### Complement, antibodies and other soluble factors

Complement activation and an increase in complement protein products correlate with severe dengue disease [47]. Large amounts of C3, C3a and C5a have been detected in DENV-infected patients and determining their levels in serum is important since these anaphylatoxins direct the lysis of infected cells and mast cell degranulation leading to histamine release [48, 49]. An increase in the number of B lymphocytes was demonstrated in DHF. [18] Total and dengue-specific IgE antibody levels were higher in patients with DHF and DSS compared with those with DF. [50] NS1 is an immunogen and high concentrations of anti-NS1 antibodies have been found in severe disease. Antibodies to NS1 can cross-react with human ECs and platelets and cause vascular permeability with production of nitric oxide (NO) and apoptosis [51, 52]. Determining the levels of dengue-specific IgE, anti-platelet and anti-EC antibodies might be used as biomarkers of severe dengue disease. Soluble factors are more stable and have the potential to serve as biomarkers. Increased levels of soluble receptors such as sTNFRII, sCD4, sCD8, sIL-2R were reported in DHF patients when compared to those with DF and that their levels correlated with disease severity [18, 53]. Release of sTNFR may be an early and specific marker of the endothelial changes that cause DSS [42]. The IL-1 receptor-like-1 protein (IL1RL1), also known as ST2, is a member of the IL1R/Toll-like receptor (TLR) superfamily. Increased serum sST2 was found in patients having secondary infection and DHF patients compared to DF patients and may be a predictive marker of dengue severity [44, 54].

## Endothelial activation markers as predictors of severe dengue disease

Endothelium is the ultimate target of permeabilizing responses and DENV influence on ECs may be direct or indirect by the release of mediators from infected or activated immune cells. DENV antigens associated with ECs were observed in autopsy samples in liver, spleen, kidney and lungs from DHF/DSS patients and infection of primary ECs by DENV has been reported [55, 56]. DENV infected ECs produce chemokine and cytokine responses that activate or recruit immune cells to the endothelium [57, 58]. Uncontrolled and persisted activation of endothelium leads to vascular permeability, microvascular thrombosis and inflammation and thus the components of activated endothelium in serum and/or plasma can serve as biomarkers of severe dengue disease [10, 59]. Under normal circumstances, the serum concentration of angiopoietin-1 (Ang-1) exceeds Ang-2, which are the mediators of endothelial function. Indonesian children showed decreased Ang-1 and increased Ang-2 levels in DHF/DSS [60]. Von Willebrand factor (vWF), a disintegrin and metalloproteinase with thrombospondin-1-like domains (ADAMTS-13) and thrombomodulin (TM) are components of coagulation system. Both vWF and ADAMTS-13 are synthesized and released by the ECs and the former stabilizes the adhesion of platelets at the site of vascular injury while the latter inhibits thrombin formation [61, 62]. Children with acute dengue infection have elevated vWF levels and the levels were particularly higher in DSS patients while ADAMTS-13 levels were decreased in children with DHF/DSS [63]. TM is present in large quantities on the surface of endothelium and acts as an anticoagulant. Serum level of sTM is proposed as diagnostic and prognostic marker of endothelial activation and dysfunction. Children from Thai and Vietnamese population show increased sTM levels particularly in children with severe dengue disease [64, 65]. Soluble forms of cell surface molecules such as sE-selectin, intercellular adhesion molecule-1 (ICAM-1) and vascular cell adhesion molecule-1 (VCAM-1) are released from ECs after activation and are used as diagnostic and prognostic markers in infectious diseases. sICAM and sVCAM-1 but not sE-selectin were found to be elevated in DSS as compared to DF and their levels do not differ between DHF and DSS [20, 66, 67]. The vascular endothelial growth factor (VEGF) is a potent stimulator of endothelial permeability and promotes proliferation, migration and survival of ECs. VEGF may contribute to inflammation and coagulation by inducing the expression of cell adhesion molecules that promotes adhesion of leucocytes. VEGFRI and VEGFRII are expressed on the ECs [68]. The VEGF and VEGFR1 plasma levels were significantly higher in DHF and DSS than those in DF while VEGFRII levels were decreased in DHF/DSS compared to DF. [28, 69] Thus, increase in Ang-2, vWF, sVCAM, VEGF and VEGFR1 levels and decrease in Ang-1, ADAMTS-13 and VEGFRII levels might serve as biomarkers of severe dengue disease.

## Biochemical markers as predictors of severe dengue disease

Several biochemical compounds are shown to be either elevated or decreased in serum/plasma of patients with severe dengue and quantifying them might serve as biomarkers of severe dengue disease. Levels of total plasma cholesterol, high-density lipoprotein (HDL) and low-density lipoprotein (LDL) were significantly decreased in children with the severest disease compared with patients with mild DHF. [70] Microbial translocation occurs during severe DENV infection and lipopolysaccharide (LPS) levels are significantly increased in dengue patients which is indicated by elevated levels of LPS binding protein (LBP) and soluble CD14 (sCD14). Elevated LPS levels in dengue patients were found to correlate with clinical disease severity [38]. Liver injury is associated with severe dengue disease with the increase in serum aspartate aminotransferase (AST), alanine aminotransferase (ALT), gamma-glutamyl transpeptidase, alkaline phosphatase, and serum albumin concentrations. Reports showed that the AST and ALT levels were high in severe disease and may serve as predictors of severe disease [32, 33, 71]. Children with DSS and liver injury have lower zinc levels and the low levels were probably caused by loss from diarrhea and from zinc translocating to liver cells [72]. The Inter-α Inhibitor Proteins (IaIp) belong to a family of serine protease inhibitors and its concentrations in pediatric patients suffering from severe DENV infection were significantly lower than in patients with mild DF and healthy controls [73]. NO is known to have a strong immunoregulatory role and in adjusting the diameter of blood vessels, remodeling blood vessels, inhibiting leukocyte adhesion, platelet aggregation, and contractile cell proliferation [74]. Serum NO levels in DHF patients were shown to be significantly lower than those of the DF patients [33, 75]. Thus, increased levels of LPS, AST, ALT and decreased levels of lipids, IaIp and NO might serve as markers of severe dengue disease.

## Host genetic markers as predictors of severe dengue disease

DNA microarrays have been used as a tool to identify genes and predict patient’s outcome for bacterial and viral infection [76]. Differential expression of genes has been shown in DF and DHF/DSS patients and hence can serve as markers of severe dengue [77, 78]. A recent study showed that analysis of seven genes (LOC286087, SLC4A4, PSPH, MYOM2, CACNA2D3, CD244 molecule, SMAD5) could possibly predict severe dengue since their expression profile was significantly lower in DHF patients compared to patients with DF. [79] DENV infection can trigger apoptosis in a variety of cell types and apoptotic cells are the main source of cell-free DNA into the circulatory system [21, 80]. Circulating DNA levels were significantly higher in patients with DENV infection than with other febrile illnesses and the increase of DNA levels can be correlated with disease severity [81].

### Conclusion

The usage of the four classes of biomarkers has advantages and limitations. Molecular markers can be accurate. However, it involves high cost for the sequencer and reagents. Immunological markers which are also seen in other inflammatory diseases require flow cytometry analysis. Although the cost of the instrument has come down, the costs of reagents are still very high for dengue endemic countries. Endothelial activation and biochemical markers are easy to estimate and the cost is low. However, the levels are modified in other disease conditions as well. Therefore, determination of combination of biomarkers will be beneficial in predicting severe dengue disease. As shown in the table 1, information on lymphocyte and platelet counts, levels of IL-10 and MIF, levels of AST and ALT, levels of gene expression and cell free DNA will aid in predicting severe dengue. With the advances in techniques and equipments, multiple biomarker analysis is possible with small amounts of patient serum samples. However, kinetics and cut-off levels need to be established particularly for children and patients in dengue endemic regions.

**Table 1:** Biomarkers of severe dengue disease

Class	Biomarker		Change	Reference
1. Immune activation markers	Cells	Plasmacytoid dendritic cells	Decrease	14
		Lymphocytes	Decrease	18, 20
		Platelets	Decrease	18, 32, 33
	Cytokines	IL-10	Increase	32, 40, 44
		MIF	Increase	39, 45
	Chemokines	CXCL-10	Increase	46
	Complements	C3a, C5a	Increase	48, 49
	Soluble receptors	sCD4/8, sIL-2R, sTNFRII	Increase	53
	Proteases	Tryptase and chymase	Increase	28
2. Endothelial activation markers	Mediators of endothelial	Angiopoietin-1	Decrease	60
	function	Angiopoietin-2	Increase	60
	Coagulation pathway	von Willebrand factor	Increase	63
	components	ADAMTS-13	Decrease	63
		sThrombomodulin	Increase	64, 65
	Cell surface adhesion molecules	sICAM, sVCAM	Increase	20, 66, 67
	Permeability mediators	VEGF, VEGFRI	Increase	28, 69
		VEGFRII	Decrease	28, 69
3. Biochemical markers	Lipids	Total cholesterol, HDL, LDL	Decrease	70
	LPS	LPB, CD14	Increase	38
	Liver enzymes	AST, ALT	Increase	32, 33, 71
	Serine protease	IaIp	Decrease	73
	Other soluble substances	Nitric oxide	Decrease	33, 75
4. Genetic markers	Gene profile	Certain gene expression	Decrease	77–79
	Circulating cell free-DNA		Increase	81

## References

[1] World Health Organization (WHO) and the Special Programme for Research and Training in Tropical Diseases (TDR) (2009). Dengue guidelines for diagnosis, treatment, prevention and control.

[2] Zhang H, Zhou YP, Peng HJ, Zhang XH, Zhou FY, Liu ZH (2014). Predictive symptoms and signs of severe dengue disease for patients with dengue fever: a meta-analysis. Biomed Res Int.

[3] Pang T, Cardosa MJ, Guzman MG (2007). Of cascades and perfect storms: the immunopathogenesis of dengue haemorrhagic fever-dengue shock syndrome (DHF/DSS). Immunol Cell Biol.

[4] Nielsen DG (2009). The relationship of interacting immunological components in dengue pathogenesis. Virol J.

[5] Perng GC, Chokephaibulkit K (2013). Immunologic hypo- or non-responder in natural dengue virus infection. J Biomed Sci.

[6] Green S, Rothman A (2006). Immunopathological mechanisms in dengue and dengue hemorrhagic fever. Curr Opin Infect Dis.

[7] Pawitan JA (2011). Dengue virus infection: predictors for severe dengue. Acta Med Indones.

[8] Dalrymple NA, Mackow ER. Roles for endothelial cells in dengue virus infection. Adv Virol. 2012;2012. 10.1155/2012/840654 PMC3431041 22952474

[9] Srikiatkhachorn A, Green S (2010). Markers of dengue disease severity. Curr Top Microbiol Immunol.

[10] Page AV, Liles WC (2013). Biomarkers of endothelial activation/dysfunction in infectious diseases. Virulence.

[11] Fink J, Gu F, Vasudevan SG (2006). Role of T cells, cytokines and antibody in dengue fever and dengue haemorrhagic fever. Rev Med Virol.

[12] Ho JJ, Wang JJ, Shaio MF, Kao CL, Chang DM, Han SW (2001). Infection of human dendritic cells by dengue virus causes cell maturation and cytokine production. J Immunol.

[13] Prestwood TR, May MM, Plummer EM, Morar MM, Yauch LE, Shresta S (2012). Trafficking and replication patterns reveal splenic macrophages as major targets of dengue virus in mice. J Virol.

[14] Pichyangkul S, Endy TP, Kalayanarooj S, Nisalak A, Yongvanitchit K, Green S (2003). A blunted blood plasmacytoid dendritic cell response to an acute systemic viral infection is associated with increased disease severity. J Immunol.

[15] De Carvalho BM, Martial J, Cabié A, Thomas L, Césaire R (2012). Decreased peripheral dendritic cell numbers in dengue virus infection. J Clin Immunol.

[16] Libraty DH, Pichyangkul S, Ajariyakhajorn C, Endy TP, Ennis FA (2001). Human dendritic cells are activated by dengue virus infection: Enhancement by gamma interferon and implications for disease pathogenesis. J Virol April.

[17] Luplerdlop N, Misse D, Bray D, Deleuze V, Gonzalez JP, Leardkamolkarn V (2006). Dengue virus infected dendritic cells trigger vascular leakage through metalloproteinase over production. EMBO Rep.

[18] Green S, Pichyangkul S, Vaughn DW, Kalayanarooj S, Nimmannitya S, Nisalak A (1999). Early CD69 expression on peripheral blood lymphocytes from children with dengue hemorrhagic fever. J Infect Dis.

[19] Azeredo EL, De Oliveira-Pinto LM, Zagne SM, Cerqueira DI, Nogueira RM, Kubelka CF (2006). NK cells, displaying early activation, cytotoxicity and adhesion molecules, are associated with mild dengue disease. Clin Exp Immunol.

[20] Azeredo EL, Zagne SM, Alvarenga AR, Nogueira RM, Kubelka CF, de Oliveira-Pinto LM. Activated peripheral lymphocytes with increased expression of cell adhesion molecules and cytotoxic markers are associated with dengue fever disease. Mem Inst Oswaldo Cruz. 2006;101:437–49. 10.1590/s0074-02762006000400016 16951817

[21] Myint KS, Endy TP, Mongkolsirichaikul D, Manomuth C, Kalayanarooj S, Vaughn DW (2006). Cellular immune activation in children with acute dengue virus infections is modulated by apoptosis. J Infect Dis.

[22] Torrentes-Carvalho A, Marinho CF, de Oliveira-Pinto LM, de Oliveira DB, Damasco PV, Cunha RV, et al. Regulation of T lymphocyte apoptotic markers is associated to cell activation during the acute phase of dengue. Immunobiology. 2014;219:329–40. 10.1016/j.imbio.2013.11.002 24508270

[23] Mongkolsapaya J, Duangchinda T, Dejnirattisai W, Vasanawathana S, Avirutnan P, Jairungsri A (2006). T cell responses in dengue hemorrhagic fever: are cross-reactive T cells suboptimal?. J Immunol.

[24] Beaumier CM, Mathew A, Bashyam HS, Rothman AL (2008). Cross-reactive memory CD8(+) T cells alter the immune response to heterologous secondary dengue virus infections in mice in a sequence-specific manner. J Infect Dis.

[25] Witczak P, Brzezińska-Błaszczyk E (2012). Mast cells in viral infections. Postepy Hig Med Dosw.

[26] King CA, Anderson R, Marshall JS (2002). Dengue virus selectively induces human mast cell chemokine production. J Virol.

[27] Brown MG, McAlpine SM, Huang YY, Haidl ID, Al-Afif A, Marshall JS (2012). RNA sensors enable human mast cell anti-viral chemokine production and IFN-mediated protection in response to antibody-enhanced dengue virus infection. PLoS One.

[28] Furuta T, Murao LA, Lan NT, Huy NT, Huong VT, Thuy TT (2012). Association of mast cell-derived VEGF and proteases in Dengue shock syndrome. PLoS Negl Trop Dis.

[29] Clark KB, Noisakran S, Onlamoon N, Hsiao HM, Roback J, Villinger F (2012). Multiploid CD61+ cells are the pre-dominant cell lineage infected during acute dengue virus infection in bone marrow. PLoS One.

[30] Sridharan A, Chen Q, Tang KF, Ooi EE, Hibberd ML, Chen J (2013). Inhibition of megakaryocyte development in the bone marrow underlies dengue virus-induced thrombocytopenia in humanized mice. J Virol.

[31] Sun DS, King CC, Huang HS, Shih YL, Lee CC, Tsai WJ (2007). Antiplatelet autoantibodies elicited by dengue virus non-structural protein 1 cause thrombocytopenia and mortality in mice. J Thromb Haemost.

[32] Malavige GN, Gomes L, Alles L, Chang T, Salimi M, Fernando S (2013). Serum IL-10 as a marker of severe dengue infection. BMC Infect Dis.

[33] Trairatvorakul P, Chongsrisawat V, Ngamvasinont D, Asawarachun D, Nantasook J, Poovorawan Y (2005). Serum nitric oxide in children with dengue infection. Asian Pac J Allergy.

[34] Juffrie M, van Der Meer GM, Hack CE, Haasnoot K, Sutaryo, Veerman AJ, et al. Inflammatory mediators in dengue virus infection in children: interleukin-8 and its relationship to neutrophil degranulation. Infect Immun. 2000;68:702–07. 10.1128/iai.68.2.702-707.2000 PMC97195 10639436

[35] Green S, Vaughn DW, Kalayanarooj S, Nimmannitya S, Suntayakorn S, Nisalak A (1999). Elevated plasma interleukin-10 levels in acute dengue correlate with disease severity. J Med Virol.

[36] Mustafa AS, Elbishbishi EA, Agarwal R, Chaturvedi UC (2001). Elevated levels of interleukin-13 and IL-18 in patients with dengue hemorrhagic fever. FEMS Immunol Med Microbiol.

[37] Pinto LM, Oliveira SA, Braga EL, Nogueira RM, Kubelka CF (1999). Increased proinflammatory cytokines (TNF-alpha and IL-6) and anti-inflammatory compounds (sTNFRp55 and sTNFRp75) in Brazilian patients during exanthematic dengue fever. Mem Inst Oswaldo Cruz.

[38] van de Weg CA, Pannuti CS, de Araújo ES, van den Ham HJ, Andeweg AC, Boas LS, et al. Microbial translocation is associated with extensive immune activation in dengue virus infected patients with severe disease. PLoS Negl Trop Dis. 2013;7:e2236. 10.1371/journal.pntd.0002236 PMC3662706 23717702

[39] Chen LC, Lei HY, Liu CC, Shiesh SC, Chen SH, Liu HS (2006). Correlation of serum levels of macrophage migration inhibitory factor with disease severity and clinical outcome in dengue patients. Am J Trop Med Hyg.

[40] Bozza FA, Cruz OG, Zagne SM, Azeredo EL, Nogueira RM, Assis EF (2008). Multiplex cytokine profile from dengue patients: MIP-1beta and IFN-gamma as predictive factors for severity. BMC Infect Dis.

[41] Rachman A, Rinaldi I (2006). Coagulopathy in dengue infection and the role of interleukin-6. Acta Med Indones.

[42] Avila-Aguero ML, Avila-Aguero CR, Um SL, Soriano-Fallas A, Cañas-Coto A, Yan SB (2004). Systemic host inflammatory and coagulation response in the Dengue virus primo-infection. Cytokine.

[43] Bethell DB, Flobbe K, Cao XT, Day NP, Pham TP, Buurman WA (1998). Pathophysiologic and prognostic role of cytokines in dengue hemorrhagic fever. J Infect Dis.

[44] Houghton-Triviño N, Salgado DM, Rodríguez JA, Bosch I, Castellanos JE (2010). Levels of soluble ST2 in serum associated with severity of dengue due to tumour necrosis factor alpha stimulation. Gen Virol.

[45] Assunção-Miranda I, Amaral FA, Bozza FA, Fagundes CT, Sousa LP, Souza DG (2010). Contribution of macrophage migration inhibitory factor to the pathogenesis of dengue virus infection. FASEB J.

[46] De-Oliveira-Pinto LM, Gandini M, Freitas LP, Siqueira MM, Marinho CF, Setúbal S (2012). Profile of circulating levels of IL-1Ra, CXCL10/IP-10, CCL4/MIP-1β and CCL2/MCP-1 in dengue fever and parvovirosis. Mem Inst Oswaldo Cruz.

[47] Nascimento EJ, Silva AM, Cordeiro MT, Brito CA, Gil LH, Braga-Neto U (2009). Alternative complement pathway deregulation is correlated with dengue severity. PLoS One.

[48] Avirutnan P, Punyadee N, Noisakran S, Komoltri C, Thiemmeca S, Auethavornanan K (2006). Vascular leakage in severe dengue virus infections: a potential role for the nonstructural viral protein NS1 and complement. J Infect Dis.

[49] Kolitha HS. Pathogenesis of Dengue Haemorrhagic Fever and Its Impact on Case Management. ISRN Infectious Diseases. 2013;2013:1-6. doi:10.5402/2013/571646

[50] Koraka P, Murgue B, Deparis X, Setiati TE, Suharti C, van Gorp EC, et al. Elevated levels of total and dengue virus specific immunoglobulin E in patients with disease of varying severity. J Med Virol. 2003;70:91–8. 10.1002/jmv.10358 12629649

[51] Saito M, Oishi K, Inoue S, Dimaano EM, Alera MT, Robles AM (2004). Association of increased platelet associated immunoglobulins with thrombocytopenia and the severity of disease in secondary dengue virus infections. Clin Exp Immunol.

[52] Lin CF, Lei HY, Shiau AL, Liu HS, Yeh TM, Chen SH (2002). Endothelial cell apoptosis induced by antibodies against dengue virus nonstructural protein 1 via production of nitric oxide. J Immunol.

[53] Kurane I, Innis BL, Nimmannitya S, Nisalak A, Meager A, Janus J, et al. Activation of T lymphocytes in dengue virus infections. High levels of soluble interleukin 2 receptor, soluble CD4, soluble CD8, interleukin 2, and interferon-g in sera of children with dengue. J Clin Invest. 1991;88:1473–80. 10.1172/JCI115457 PMC295652 1939640

[54] Becerra A, Warke RV, de Bosch N, Rothman AL, Bosch I (2008). Elevated levels of soluble ST2 protein in dengue virus infected patients. Cytokine.

[55] Jessie K, Fong MY, Devi S, Lam SK, Wong KT (2004). Localization of dengue virus in naturally infected human tissues, by immunohistochemistry and in situ hybridization. J Infect Dis.

[56] Bosch I, Xhaja K, Estevez L, Raines G, Melichar H, Warke RV (2002). Increased production of interleukin-8 in primary human monocytes and in human epithelial and endothelial cell lines after dengue virus challenge. J Virol.

[57] Avirutnan P, Malasit P, Seliger B, Bhakdi S, Husmann M (1998). Dengue virus infection of human endothelial cells leads to chemokine production, complement activation, and apoptosis. J Immunol.

[58] Huang YH, Lei HY, Liu HS, Lin YS, Liu CC, Yeh TM (2000). Dengue virus infects human endothelial cells and induces IL-6 and IL-8 production. Am J Trop Med Hyg.

[59] Lee WL, Liles WC (2011). Endothelial activation, dysfunction and permeability during severe infections. Curr Opin Hematol.

[60] Michels M, van der Ven AJ, Djamiatun K, Fijnheer R, de Groot PG, Griffioen AW, et al. Imbalance of angiopoietin-1 and angiopoetin-2 in severe dengue and relationship with thrombocytopenia, endothelial activation, and vascular stability. Am J Trop Med Hyg. 2012;87:943–6. 10.4269/ajtmh.2012.12-0020 PMC3516273 22949515

[61] Lowenstein CJ, Morrell CN, Yamakuchi M (2005). Regulation of Weibel-Palade body exocytosis. Trends Cardiovasc Med.

[62] Tati R, Kristoffersson AC, Ståhl AL, Mörgelin M, Motto D, Satchell S (2011). Phenotypic expression of ADAMTS13 in glomerular endothelial cells. PLoS One.

[63] Djamiatun K, van der Ven AJAM, de Groot PG, Faradz SM, Hapsari D, Dolmans WM, et al. Severe dengue is associated with consumption of von Willebrand factor and its cleaving enzyme ADAMTS-13. PLoS Negl Trop Dis. 2012;6:e1628. 10.1371/journal.pntd.0001628 PMC3341341 22563509

[64] Butthep P, Chunhakan S, Tangnararatchakit K, Yoksan S, Pattanapanyasat K, Chuansumrit A (2006). Elevated soluble thrombomodulin in the febrile stage related to patients at risk for dengue shock syndrome. Pediatr Infect Dis J.

[65] Wills BA, Oragui EE, Stephens AC, Daramola OA, Dung NM, Loan HT (2002). Coagulation abnormalities in dengue hemorrhagic Fever: serial investigations in 167 Vietnamese children with Dengue shock syndrome. Clin Infect Dis.

[66] Koraka P, Murgue B, Deparis X, Van Gorp EC, Setiati TE, Osterhaus AD, et al. Elevation of soluble VCAM-1 plasma levels in children with acute dengue virus infection of varying severity. J Med Virol. 2004;72:445–50. 10.1002/jmv.20007 14748068

[67] Khongphatthanayothin A, Phumaphuti P, Thongchaiprasit K, Poovorawan Y (2006). Serum levels of sICAM-1 and sE-selectin in patients with dengue virus infection. Jpn J Infect Dis.

[68] Shibuya M, Ito N, Claesson-Welsh L (1999). Structure and function of vascular endothelial growth factor receptor-1 and −2. Curr Top Microbiol Immunol.

[69] van de Weg CA, Pannuti CS, van den Ham HJ, de Araújo ES, Boas LS, Felix AC, et al. Serum angiopoietin-2 and soluble VEGF receptor 2 are surrogate markers for plasma leakage in patients with acute dengue virus infection. J Clin Virol. 2014;60:328–35. 10.1016/j.jcv.2014.05.001 24928471

[70] van Gorp EC, Suharti C, Mairuhu AT, Dolmans WM, van Der Ven J, Demacker PN, et al. Changes in the plasma lipid profile as a potential predictor of clinical outcome in dengue hemorrhagic fever. Clin Infect Dis. 2002;34:1150–3. 10.1086/339539 11915007

[71] de Souza LJ, Nogueira RM, Soares LC, Soares CE, Ribas BF, Alves FP (2007). The impact of dengue on liver function as evaluated by aminotransferase levels. Braz J Infect Dis.

[72] Laoprasopwattana K, Tangcheewawatthanakul C, Tunyapanit W, Sangthong R (2013). Is zinc concentration in toxic phase plasma related to dengue severity and level of transaminases?. PLoS Negl Trop Dis.

[73] Koraka P, Lim YP, Shin MD, Setiati TE, Mairuhu AT, van Gorp EC, et al. Plasma levels of inter-alpha inhibitor proteins in children with acute Dengue virus infection. PLoS One. 2010;5:e9967. 10.1371/journal.pone.0009967 PMC2850310 20386596

[74] Chaturvedi UC, Nagar R (2009). Nitric oxide in dengue and dengue haemorrhagic fever: necessity or nuisance?. FEMS Immunol Med Microbiol.

[75] Valero N, Espina LM, A˜nez G, Torres E, Mosquera JA (2002). Short report: increased level of serum nitric oxide in patients with dengue. Am J Trop Med Hyg.

[76] Ramilo O, Allman W, Chung W, Mejias A, Ardura M, Glaser C (2007). Gene expression patterns in blood leukocytes discriminate patients with acute infections. Blood.

[77] Nascimento EJ, Braga-Neto U, Calzavara-Silva CE, Gomes AL, Abath FG, Brito CA, et al. Gene expression profiling during early acute febrile stage of dengue infection can predict the disease outcome. PLoS One. 2009;4:e7892. 10.1371/journal.pone.0007892 PMC2775946 19936257

[78] Ubol S, Masrinoul P, Chaijaruwanich J, Kalayanarooj S, Charoensirisuthikul T, Kasisith J. Differences in global gene expression in peripheral blood mononuclear cells indicate a significant role of the innate responses in progression of dengue fever but not dengue hemorrhagic fever. J Infect Dis. 2008;197:1459–67. 10.1086/587699 18444802

[79] Sun P, García J, Comach G, Vahey MT, Wang Z, Forshey BM, et al. Sequential waves of gene expression in patients with clinically defined dengue illnesses reveal subtle disease phases and predict disease severity. PLoS Negl Trop Dis. 2013;7:e2298. 10.1371/journal.pntd.0002298 PMC3708824 23875036

[80] Lichtenstein AV, Melkonyan HS, Tomei LD, Umansky SR (2001). Circulating nucleic acids and apoptosis. Ann N Y Acad Sci.

[81] Ha TT, Huy NT, Murao LA, Lan NT, Thuy TT, Tuan HM (2011). Elevated levels of cell-free circulating DNA in patients with acute dengue virus infection. PLoS One.

